# Supplementary material for: Exploring the Maintaining Period and the Differentially Expressed Genes between the Yellow and Black Stripes of the Juvenile Stripe in the Offspring of Wild Boar and Duroc
Source: Animals (Basel). 2024 Jul 19;14(14):2109. doi: 10.3390/ani14142109 (PMC11274008; doi:10.3390/ani14142109)
Supplement: Supplementary file 1 [file animals-14-02109-s001.zip › animals-3103931-supplementary figures.pdf]

Supplementary figures

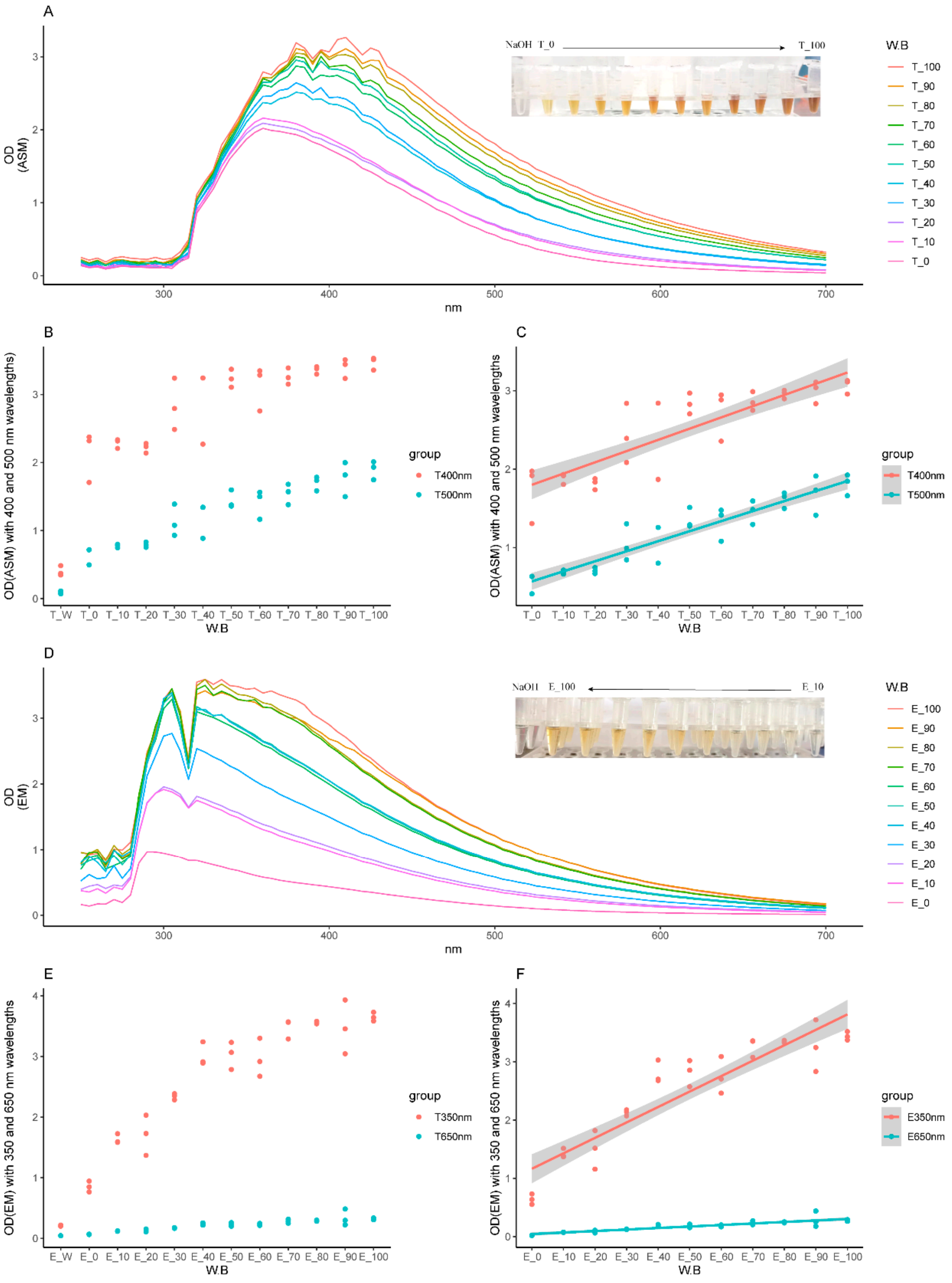

Figure S1. Optical density (OD) values of alkaline soluble melanin (ASM) and eumelanin (EM) with different wave lengths and black hair proportions. **(A)** ASM with wave lengths from 250 to 700nm; **(B)**and **(C)**. ASM with 400 and 500 nm wave lengths; **(D).** EM with wave lengths from 250 to 700nm; **(E)** and **(F)**. EM with 350and 650 nm wave lengths. W.B refers to the weight of black hair powder.

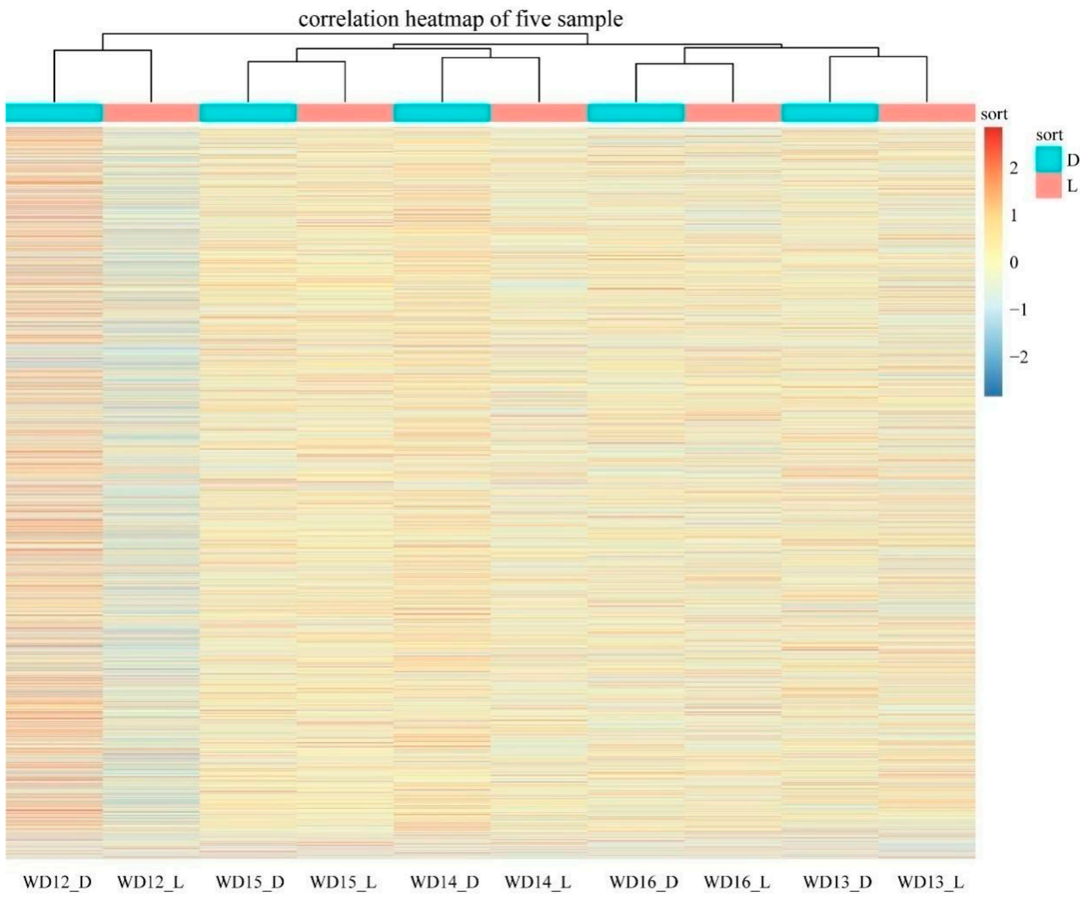

Figure S2. The gene expression profiles of all samples

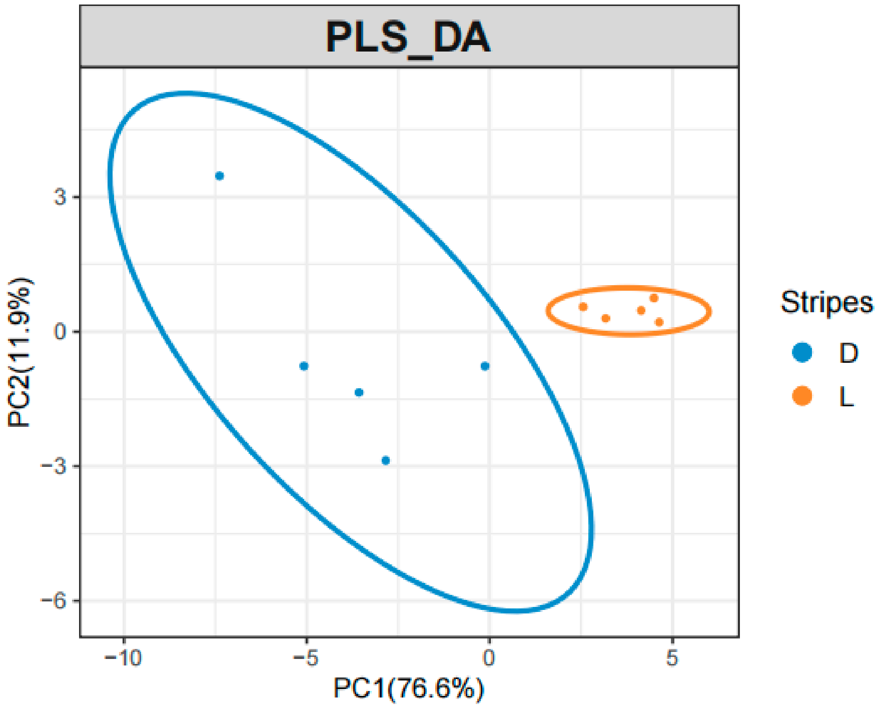

Figure S3. PLS-DA analysis for DE-mRNAs and DE-lncRNAs between the black and yellow stripes.
